# Co-creative art processes with cancer patients from the artists’ perspective: a qualitative study exploring resonance theory

**DOI:** 10.1007/s00520-023-07744-0

**Published:** 2023-04-20

**Authors:** Yvonne Weeseman, Michael Scherer-Rath, Nirav Christophe, Henny Dörr, Esther Helmich, Mirjam A. G. Sprangers, Niels van Poecke, Hanneke W. M. van Laarhoven

**Affiliations:** 1 Amsterdam University Medical Centers, Department of Medical Oncology, University of Amsterdam, De Boelelaan 1117, Amsterdam, The Netherlands; 2 Treatment and Quality of Life, Cancer Center Amsterdam, Amsterdam, The Netherlands; 3 Faculty of Philosophy, Theology and Religious Studies, Radboud University, Nijmegen, The Netherlands; 4 HKU University of the Arts Utrecht, Utrecht, The Netherlands; 5 Amsta Healthcare Organization, Amsterdam, The Netherlands; 6 Medical Psychology, Amsterdam UMC Location University of Amsterdam, Amsterdam, The Netherlands; 7 Mental Health, Amsterdam Public Health, Amsterdam, The Netherlands

**Keywords:** Supportive care, Advanced cancer patients, Co-creation, Life events, Resonance theory, Quality of life

## Abstract

**Purpose:**

Co-creation, characterised by artists and patients creating a joint work of art, may support patients with the integration of life events into their life story, such as living with cancer. In the process of co-creation, resonance relationships between patients, artists and material may evolve that support integration. We aim to investigate if and if so, how resonance relationships occur from the perspective of the artist.

**Methods:**

We used the first 10 audio recordings of supervision sessions between eight artists and their two supervisors on ongoing co-creation processes with cancer patients. By conducting a qualitative template analysis in AtlasTi, we searched for the presence of resonance, as defined by its four main characteristics, *Being affected, touched and moved*; *Self-efficacy and responding*; *Moments of uncontrollability*; and *Adaptive transformation*. In addition, two case descriptions are presented.

**Results:**

We found resonance relationships to be present in the studied co-creation processes where moments of uncontrollability can lead to a next step in the process of co-creation and as such form an important factor within co-creation.

**Conclusions:**

The current study suggests focus on elements of resonance relationships within co-creation, specifically practising with uncontrollability while working with art, could strengthen interventions targeting integration of life events in advanced cancer patients.

## Introduction

The experience of living with cancer can reduce quality of life of patients [1]. Interventions aiming to support patients in enhancing quality of life could include art, for instance as art therapy [2–7], or, more recently, as co-creative processes [8–11]. Art therapy tends to have a more therapeutic focus [12] compared to co-creation [8]. In co-creation, an artist supports patients to express aspects of the patient’s life narrative, culminating into a work of art [8–11]. Co-creation is characterised by the use of multiple senses and imagination, supporting various ways of expression in patients [9, 12, 13]. The experiences that are addressed could include a sense of loss, uncontrollability, uncertainty or despair, all related to an inability to control the circumstances patients are facing [8, 9, 14]. An example of co-creation is creative writing where a patient, together with an artist, expresses her feelings of being overwhelmed by her cancer and its treatment.

In previous studies on co-creation, artists indicated that the relation between patient and both artist and material is of a reciprocal nature and forms an important factor facilitating the co-creation process. Artists suggested that the co-creation process only becomes meaningful when patients become engaged [8, 11]. Another factor in co-creation processes with patients seems to include a confrontation with circumstances beyond one’s control which affect the patient and create a meaningful action perspective for placing the experience within their life narrative [8, 11, 15].

The reciprocal nature of relations which actors have with their worlds is acknowledged by theories on resonance [16, 17], as well as by certain orientations within psychotherapy [18, 19]. Yet, the factor of uncertainty due to experiences beyond one’s control is either not well described, as in general theories on resonance [16], or viewed as unfavourable, when the aim of self-efficacy [19] or coping [20] partly lies in regaining a sense of control over one’s existence. Contrary to the above [16, 19, 20], resonance theory by Rosa [21] describes that the core of a resonance relationship lies in its indefinite quality and attuned appeal which creates an immediate, intense, embodied, sounding relationship between the person, the other(s) and the tangible world. Uncontrollability is an ever present characteristic of these relationships. Rosa’s theoretical assumptions seem particularly important for co-creation processes as resonance theory describes how people are affected by others or by the world, how they respond and how they are subsequently changed, and, as such, could deepen our understanding of how patients are affected and changed during co-creation processes.

A resonance process may unfold when one is sufficiently responsive to others, oneself and the world. An open attitude, the ability to be affected and the flexibility to change one’s perspective are prerequisites to experience resonance [21]. Resonance has been described to have four main characteristics.

(1) *Being affected*, touched or moved by the world is the first experience of a subject, which may stimulate an intrinsic interest in that which affects him or her. This intrinsic interest comes forth when one’s core values are affected in contact with the world, which becomes evident through bodily sensations, emotions and thoughts. (2) *Self-efficacy* unfolds when the person is able to actively reciprocate the call of that which affected him or her. (3) *Uncontrollability* is the element that is ingrained in the full experience of resonance. We cannot know when and how the world is going to affect us, nor do we know beforehand how to relate to being affected by the world. If one does not have the ability to be open to new experiences or to the unexpected, there is no possibility for resonance. (4) *Adaptive transformation* takes place when the person who is affected is able to respond effectively, which in turn changes both the person’s experience in the encounter with the unexpected as well as the person him or her-self.

According to Rosa, relations that contain these four main characteristics of resonance affect and change those who have these relations [21, 22]. In line with Rosa, here we will call these relations ‘resonance relationships’. As far as we know, no studies have been published yet that further investigate the nature of these relations in a co-creation setting.

In this study, we will first investigate, if and if so, how resonance relationships, as defined by their four main characteristics, occur within co-creation processes, from the perspective of the artist. Secondly, we will investigate how artists use these resonance relationships within co-creation.

## Methods

### Participants

For the current study, we interviewed professional artists who are participating in the In Search Of Stories project (ISOS) [23]. ISOS, funded by the Dutch Cancer Society, is an ongoing project evaluating a narrative multimodal intervention aimed at enhancing quality of life in palliative cancer patients recruited from four Dutch hospitals [23]. Within ISOS, a spiritual counsellor supports patients with a life review interview, drawing of a rich picture [24] and reading selected literature [23], followed by a co-creation process supported by a professional artists [11]. The artists involved in ISOS meet two inclusion criteria: extensive experience in co-creation processes with patients and the ability to work with a broad repertoire of art modalities in addition to their main art modality, thereby enhancing their possibilities to support patients during the co-creation processes. The ISOS project includes 11 artists with experience in a variety of primary art forms who are all eligible for the current study, for further details see [11]. Within the ISOS project, individual artists reflect on their ongoing co-creation processes with patients, together with two supervisors. Both supervisors are senior faculty members of the HKU University of the Arts Utrecht. Supervision sessions with the artist and both supervisors take place one to three times during a co-creation process. Supervision sessions can focus on the process and patient, but also on logistics and provision of the artistic material used in the co-creation process, or on future presentations. Supervision sessions are intended to support the artist during co-creation processes and all supervision sessions are audio recorded.

### Study design and data collection

We used a qualitative study design in which we audio recorded supervision sessions between professional artists and their supervisors as they reflected on co-creation processes with palliative cancer patients within the ISOS project. Artists and supervisors were not trained in, or familiar with resonance theory, nor were the supervision sessions based on resonance theory.

### Data analyses

We have first used template analysis to search for the presence and content of characteristics of resonance relationships within our study material. Secondly, we have used directed content analysis of specific sections of our study material to investigate how artists use resonance relationships within co-creation. All presented quotations and constructs were translated from Dutch into English by YW.

To investigate our first research question—if and if so, how resonance relationships occur within co-creation processes—we looked for the presence and content of the four main characteristics of resonance by means of template analysis [25, 26]. Template analyses offer steps of analyses which clarify how a conceptual model can be developed and deepened by a comparison and elaboration of the collected empirical data. Template analyses entail several rounds of familiarising oneself with the data, identification, labelling and coding of themes and categories, organising emerging themes into larger wholes of meaningful clusters and categories, until all data are integrated in the emerging template, culminating in a final template. The main procedural steps of the analyses of our audio recordings are shown in Table 1. The audio recordings of the supervision sessions were imported in AtlasTi [25]. AtlasTi offers a possibility to directly analyse audio recordings, instead of plain text, which adds details of tonality and intonation of the spoken words into the analyses. The audio recordings were primarily analysed by YW (MA, MSc, MSc, female), who has a professional background in art therapy, clinical psychology and spiritual care. Within template analyses, so-called a priori themes can be identified based on literature, current knowledge or experience [26, 27]. These a priori themes serve as a preliminary template. During the data analyses, the preliminary template can be adjusted or changed to fit all relevant data until saturation is reached, culminating in the final template [26, 27]. Our preliminary template was based on the four main characteristics of the theoretical concept of resonance (see Table 2) [21]. Results of the data analyses were discussed with MSR (Associate professor, PhD, male), who has a professional background in religious studies, theology, spiritual care, template analyses and qualitative research. The final template was discussed with MSR and HvL (Professor, MD, PhD, PhD, female), who has a professional background in medical oncology and theology.

**Table 1:** Main procedural steps of the data analyses

**Fig. 1:**
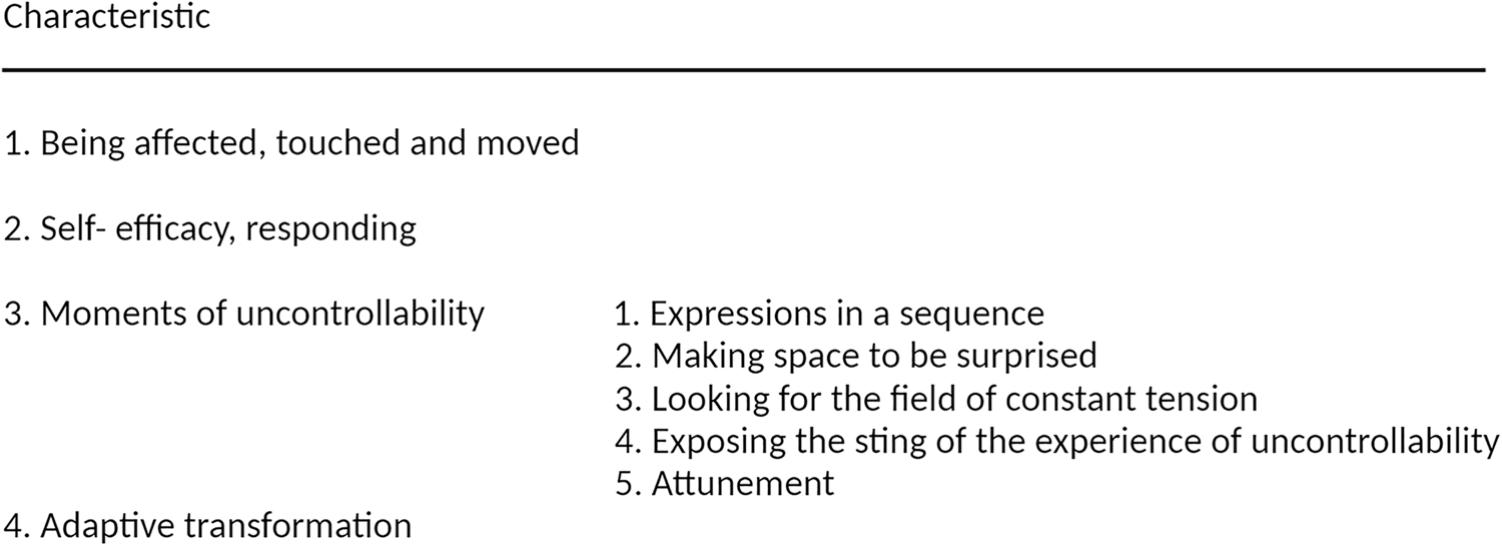
Final template of the characteristics of resonance

1. Four a priori themes based on resonance theory were identified by YW and discussed in detail with MSR and HvL. These a priori themes were labelled ‘being affected’, ‘self-efficacy’, ‘uncontrollability’ and ‘adaptive transformation’
2. For data analyses, the a priori themes, ‘being affected’, ‘self-efficacy’, ‘uncontrollability’ and ‘adaptive transformation’, were included in the preliminary template (see Table 2)
3. YW familiarised herself with the supervision session data by careful listening and extensive re-listening of the first three supervision sessions
4. The first three supervision sessions were analysed by YW. While using the preliminary template, YW analysed the imported data of the first three supervision sessions and themes and categories were distinguished and subsequently labelled with a code. If needed a priori themes were adapted or confirmed and new emerging themes were identified, defined and included
5. Encompassing the preliminary understanding of the relationships between and within themes, YW organised the emerging themes into larger wholes of meaningful clusters and categories
6. YW translated the results of this preliminary analysis into a secondary template. The secondary template contained the tentative themes and codes of the first three supervision sessions. The secondary template was discussed with MSR
7. The a priori themes of the preliminary template were confirmed in the secondary template: ‘being affected’, ‘self-efficacy’, ‘uncontrollability’ and ‘adaptive transformation’
8. Subsequently, YW familiarised herself with the data of the remaining seven supervision sessions by careful listening and extensive re-listening of these seven supervision sessions
9. Following the secondary template, YW analysed the final seven supervision sessions
10. The resulting third template, based on seven supervision sessions, confirmed the secondary template for the themes ‘being affected’, ‘self-efficacy’ and ‘adaptive transformation’, but differed for the theme ‘uncontrollability’. The theme ‘uncontrollability’ was multi-layered and better defined by five subthemes: ‘expressions in a sequence’, ‘making space to be surprised’, ‘looking for the field of constant tension’, ‘exposing the sting of the experience of uncontrollability’ and ‘attunement’
11. Subsequently, the full set of 10 supervision sessions was re-analysed by YW in an iterative process where the template was continuously accommodated to contain all themes and codes. Themes and codes were modified to better represent the data. This iterative process continued until all recorded data were mapped onto the template. Analysis indicated data saturation and no elements contradicting the concepts based on resonance theory were identified in the data
12. Finally, themes, codes and categories were grouped into concepts and constructs forming the final template which contained all themes and codes. The final template was discussed with MSR and HvL. The final template resembled and confirmed the third template. Some labels of the characteristics were slightly extended to better encompass their content. The four themes were ‘being affected, touched and moved’, ‘self-efficacy, responding’, ‘moments of uncontrollability’ and ‘adaptive transformation’. The theme ‘moments of uncontrollability’ was further specified and subdivided in five subthemes: ‘expressions in a sequence’, ‘making space to be surprised’, ‘looking for the field of constant tension’, ‘exposing the sting of the experience of uncontrollability’ and ‘attunement’. The final template is shown in Fig. 1

**Table 2:** Characteristics of resonance and their descriptions as used in the preliminary template

Characteristic	Descriptions
*1. Being affected*	The first moment of unexpectedness when the subject is affected by the world, in other words, is touched or moved in such a way as to develop an intrinsic interest in the segment of the world encountered and to feel somehow addressed by it
*2. Self-efficacy*	The affected person is challenged to actively react and reciprocate the call of that which moved him or her
*3. Uncontrollability*	The experience of not knowing when and how the world is going to affect us, or not knowing beforehand how to relate to the experience. Unexpectedness is ingrained in the full experience of resonance
*4. Adaptive transformation*	A change in the person induced by experiencing the encounter with the unexpected. Being affected will add something new to the experience and can lead to a different perspective on the circumstances. The new perspective is intrinsically connected to existential values and experiences, but also generates new energy to welcome newness and different thoughts and feelings

#### Case descriptions

To investigate our second research question, how artists use resonance relationships within co-creation, and to deepen our insights from investigating our initial research question, completed co-creation processes were selected. Co-creation processes from start to finish illustrate the full temporal developments within resonance relationships. The audio recordings of the supervision sessions of these cases were analysed by means of directed content analysis [28, 29]. Directed content analysis is a deductive approach suitable to analyse a text for an already established framework of concepts [28, 29]. Here we used the framework which had been established in our analyses of the characteristics of resonance relationships. YW analysed the data focussing on the presence of the themes of the final template in combination with temporal developments in both the main characteristics of resonance relationships and, more importantly, on how artists use these resonance relationships within co-creation. All analyses were conducted in AtlasTi and discussed with MSR and HvL.

### Ethics

The study was performed in line with the principles of the Declaration of Helsinki. This study was exempted from ethical approval by the Medical Ethics Review Committee of the Academic Medical Centre, since the Medical Research Involving Human Subjects Act was not applicable (reference number: W20_436 # 20.483). Written informed consent from every participating patient was obtained at the start of their enrolment. The professional artists interviewed for the current study were employed within the ISOS project and therefore informed consent from them was not applicable.

## Results

### Participants and setting

In total, ten supervision sessions focussing on the co-creation process with the patient, involving eight of the eleven artists and two supervisors, were available at the time of the study. The other three artists were not yet involved in a co-creation process within the ISOS project. The eight participating artists were musicians, visual artists, scenographers, a theatre composer and a creative writer (see Table 3). The supervision sessions were held at the HKU University of the Arts Utrecht. Supervision sessions took place between April 2021 and August 2021. The duration of the supervision sessions varied between 30 and 60 min, with a median duration of 45 min.

**Table 3:** Participating artists involved in the co-creation processes

Field of expertise	Age	Gender
A: Musician	42	Male
B: Musician	38	Male
C: Visual artist^1^	39	Female
D: Visual artist^2^	51	Female
E: Scenographer	50	Female
F: Scenographer	49	Female
G: Theatre composer^1,2^	50	Female
H: Creative writer	58	Female

^1^The case descriptions are based on the audio recordings of the supervision sessions of artists C and G

^2^For artists D and G, two supervision sessions were analysed

### Characteristics of resonance relationships

To investigate our first research question—if and if so, how resonance relationships occur within co-creation processes—we first looked for the presence of the characteristics of resonance, which are described in the final template (see Fig. 1), and secondly, these characteristics are described in more detail, including artist’s quotations, in Table 4.

**Table 4:** Characteristics of resonance relationships in the process of co-creation, with artist’s quotations

1. Being affected, touched and moved For the artists, colour, texture, mass, format and movement offered numerous possibilities to evoke an inner experience in the patient. Artists reported to have shown the patient images that activated visual impressions or initiated a silent walk to listen to sounds, which activated the auditory senses. Attention could also be focussed on awareness of the fingertips, which stimulated sensibility through touch. Artists also used the environment to set up a rich space of possibilities to meet the ‘world’. To exemplify, they invited patients into their art or sound studio, they went to special places in nature, or they went on short trips in a designer campervan	*I invited him into my sound studio and he immediately picked up an electric guitar and started to play some melancholic tunes. I started to add some synthesizer sounds from the sixties to his tunes to make a fuller sound, within a few minutes we had some music going. (B)*^1^ *I had her close her eyes and picked some fabrics and shapes from what was laying around in my studio anyway. I placed it in her hands and asked her to sense and see if this would allow new associations and stories to arise based on what she was feeling in her hands. (E)* *From my expertise in drawing it is interesting to see what images she has in mind around the anger she experiences towards her doctors, and I am totally open to not judge any of it, I just want to see how we can create a space to fully dive into her imagination, and subsequently reflect on that together. (D)*
2. Self-efficacy, responding Artists reported that patients experienced recognition in the creation of art. For example, a white blanket was being painted with flowers representing a patient’s uterus containing a tumour. The blanket both expressed a need for comfort and a recognition of the illness being part of the patient. In general, patients responded to the material by trying to adapt, manipulate and edit it according to their vision of what they wanted to create. Within this process, they encountered difficulties in modelling the material, challenging them to actively respond to the material. The artists facilitated input by sharing their thoughts on the created work. Artist and patient were in a constant interaction to negotiate the next step	*There are certain elements she insists need to be added to her book and she wants to describe her illness story in a linear way. I have made quite a few suggestions, some things she responds to, others she ignores. There is an option to add a dialogue between her and her husband about what was, and could have been, but she is very determined. She wants to stay mostly with facts and focussed on writing an autobiography about the course of her illness. (H)* *He had made a drawing of a road which leads to the horizon, towards the light, while at the left of the road a thunderstorm is raging. We used synthesizers to create the sound of thunder. He then wanted to work on this piece with his own amateur equipment at home to create additional tunes to add to the underlying bleak feeling of the sound. (A)*
3. Moments of uncontrollability	
*Expressions in a sequence* The artists indicated that they stimulated the patients to become aware of the sequence of their successive experiences within the co-creation process and how these experiences were expressed within the work of art. The consecutive expression of experiences illustrated that meaning can change over time and is not fixed. The artists also indicated that they wanted the patient to experience those different perspectives exist at the same time. Without different perspectives, no movement was possible and the changeability ceased to exist	*She wants a sequence of events in her book, so we are making a structure in which variety is combined with linearity. This includes facts about cancer in general, a letter, pictures, but also memories. The last time she was drunk and danced like crazy is described in detail, that was three days before she was diagnosed. In this way the different elements and perspectives together make up the whole. (H)*
*Making space to be surprised* Artists also emphasised to have made space for the patient to experience that their expressions during their creation process was not representing them completely; the patient was always more than what they tried to express. The artists reported that they wanted to inspire the patient to keep all options open and to let the patient experience that everything is possible during the creation process. The artists invited the patient to follow their own interests, desires and curiosities, meanwhile bringing forward their own thoughts, even if these were opposing the patient’s ideas	*I am next planning for making a trip with my campervan to a special place out in nature and then present her the table I made with ten distinct objects. She can ascribe her own meaning to them and create a storyline. It will become a minitheater with objects that can change over time. I feel I want to surprise her with this idea as part of my artistic input. (F)*
*Looking for the field of constant tension* The artists explained they were looking for constant tension without overtly explaining this to the patient. When choosing for a particular medium, they would, for example, try to postpone the choice of what the end result would be, provoked the patient to be more detailed about his/her experience, or they would deliberately not directly go to the core of a problem but worked around it to keep a high amount of tension throughout the process of art making	*I let myself be instructed by her as I keep giving input. I ask for a an extreme level of detail when she talks about her experience. Working this way she has to open up to the felt sense of feeling spheres of tension when objects are just off balance when they are placed together, and don’t exactly match her experience. (C)* *I notice a strong urge with her to want to know exactly what the end result will be, just like the tendency to control her illness. She is quite outspoken about anything and that makes me have the tendency to stay away from concrete planning, and instead make space to explore. She says she has no musical taste, but that is very interesting to research a bit more, maybe she does listen to music and went to concerts in the past. The question remains when to follow her in her ways, or when to challenge her. (A)*
*Exposing the sting of the experience of uncontrollability* Artists said that the aesthetic work of art contained emotional pain, because it is a reflection of the struggle with the experience of uncontrollability that could not be solved. For example, this work of art could be an anger monologue towards healthcare professionals in an autobiographical story on the experience of cancer, or a coloured necklace made out of ‘used’ bandage, which represented treatment in hospital. In all these works of art, the pain had to be incorporated representing something that cannot disappear	*She has the tendency to look for the optimistic part of the experiences, but I keep asking her if the pain can also be included. I ask her: “Image you will be dead in some years, where is this artwork going to be? Is it in your windowsill, or on your grave? Should it be dismantled so all your loved ones can receive a part of it, or should it be opened up so your ashes can be stored in it?” I know I am provoking, and I don’t say: “Imagine being dead within a month”, but I want it to be completely complete, containing alle her feelings. And that’s why I want to express her needs, so we can fit these too within the work. (C)* *She is furious about the way the diagnostic process was hindered, and wants to write an anger-monologue. I help her to write straight from her feelings like “I have tears in my eyes” instead of “I feel anger” and when it doesn’t work to write because the feelings are too strong, I ask her to write about the sensations in her body, for example “I feel sick”, to give her the experience to write from her felt sense. (H)*
*Attunement* Artists used the word attunement to describe the level of resonance within resonance relationships. Also, artists reported they consciously and actively supported high levels of attunement. High levels of attunement between artist and patient contributed to a safe space for the patient, allowing deeper emotions connected to the experience of uncontrollability. They said that in this way the sting of uncontrollability could be felt while the patient simultaneously had sufficient resilience to allow this experience to exist and not resist it. A temporary lack of attunement between artist and patient and/or between patient and material evoked further struggles in the patient, which could support the next step in the co-creation of the work of art. Artist reported they modulated the level of attunement and thereby the intensity of the resonance in the resonance relationships between patient and both material and artist, which affected the intensity of the emotions the patient felt and the intensity of the experience of the patient while working with the material during the co-creation process	*Our collaboration was getting more productive once we could add more internal voices to the palette, not just the dominant voices, but waking up different perspectives. Our starting question could be “what is it that we want to discover together?” And together we are looking for what is important and needs to be expressed. In this way the artwork, but also the process will become more interesting. (D)* *I am not looking for friction, when we rewrite sentences that are very dear to her, I ask her to zoom into those sentences and add what she feels physically. At that point I push it a bit, and that could create some tension. But if she really wants to conserve certain sentences, that’s fine and we keep it that way. And we agreed to both speak out what is important to us and that helps in the process. (H)*
4. Adaptive transformation Artists indicated that the patient and artist were both changed by the experience of art making. The patient witnessed the changes in the work of art, which was found to stimulate further change, and mirrored the inner experience of the patient at the same time. Throughout the co-creation process many different perspectives were creatively explored and each phase of the process was valuable because it exposed the patient’s experiences and enabled the patient to assimilate the experiences into their identity. For example, one patient expressed his experience with cancer by producing a piece of music with a side A (the brighter side) and a side B (the dark side). This work of art became a ‘keeper of experiences’, which reflected a perspective that changed each time the patient listened to it, providing him the opportunity to connect both sides of his experience. He was thus invited to creatively perceive his own narrative from another angle and to transform his inner world. Artists spoke about multiple rounds of creative exploration and discovery as an iterative process, where after each preliminary interpretation a new perspective unfolded	*During the process she does give the course of her illness a meaningful place. She says: “Half a year ago I thought I would be dead by now, but now as I am also receiving clear scans again I am more confident to create a bit of future once again.” (H)* *He asked me how I experience working with him. When I told him about what cancer means to me, he allowed a shift from always talking in an optimistic way about his cancer, towards talking about deeper feelings and doubts that he secretly also experiences. (B)*

^1^See Table 3 for information on artist

#### Final template

The final template mainly resembled the preliminary template; however, uncontrollability showed to be multi-layered and included several distinct dynamics and mechanisms. Therefore, the characteristic *Moments of uncontrollability* was further subdivided into *Expressions in a sequence*, *Making space to be surprised*, *Looking for the field of constant tension*, *Exposing the sting of the experience of uncontrollability* and *Attunement*. Some labels of the characteristics were slightly extended to better encompass their content. The final template is shown in Fig. 1.

#### Characteristics of resonance relationships in the process of co-creation

The characteristics of resonance relationships are described in detail and illustrated with artist’s quotations in Table 4. Artists are referred to with a capital letter, corresponding to Table 3.

### Case descriptions

Two cases are described to illustrate the temporal aspect of our findings on the second part of our first research question, how resonance relationships, as defined by their four main characteristics, occur within co-creation, and, more importantly, to answer our second research question, how artists use these resonance relationships within co-creation. These two cases are examples of different approaches in resonance relations that can be used by artists in co-creation processes. During the co-creation process, resonance relationships can provide a point of focus to alternate with moments of uncontrollability. Artists kept the patient interested and connected to the co-creation process during moments of uncontrollability by focussing on either or both resonance relationships. In case one, the artist puts more emphasis on the resonance relationship between patient and artist, whereas in case two, the artist puts more emphasis the resonance relationship between patient and material (Table 5).

**Table 5:** Case descriptions of resonance relationships, with artist’s quotations

Case one. Characteristics of resonance and emphasis on the resonance relationship between patient and artist during moments of uncontrollability This 50-year-old female patient was undergoing immunotherapy for cervical cancer. As part of the ISOS intervention, she had chosen to read a story called ‘The ant’s departure’ written by Toon Tellegen, in which the protagonist of the story, the ant, suddenly disappears and the other animals in the woods are left wondering what has happened to the ant [23]. She was very much touched by the story and identified with the ant as a reflection of herself. She projected on the animals in the story visions of her own family members, as they were wondering where the ant could have gone to, as it suddenly disappeared from the woods. She had been worrying about how her family members would handle her eventual departure. Prior to the first co-creation session, she had a dream in which she had seen herself being a solid rock in wild waters in relation to her family. She wanted to create this rock combined with the word ‘me’, which captured her most prominent identity in life. She felt a light would have to shine from within the rock reflecting her inner strength. This light should not be visible in daylight, but would only be discernible when darkness would fall	
*Characteristic 1: Being affected, touched and moved* At the start of the process, the artist offered various materials to invite the patient to relate to its mass, format and texture. She subsequently probed the patient to connect to the material and to become affected by the material as they started creating the rock. The artist was aware that making the rock would include working with hard and soft textures during the various rounds of several artistic creations, as well as that different materials would evoke a different reaction in the patient	*I invited her to look for pictures of specific rocks and then I showed her different materials that could be used in the creation process of her rock. These materials were introduced during several rounds of artistic creation, starting with making several samples of clay to find the right shape of the rock. For the actual creation of the rock, we used chicken wire to create the framework, then non-woven wallpaper to place a lining over the chicken wire, and finally paper mâché to create the right transparency. These materials each had their own distinct quality and I tried to invite the patient to feel their texture and possibilities to sense if it “felt right” while simultaneously having the aim of creating this rock in the back of her mind*
*Characteristic 2: Self-efficacy, responding* Within this art making process, self-efficacy was most prominent in the struggle the patient experienced to control the material, especially in combination with her high standards. The artist chose the strategy of ‘not knowing’ and did not provide suggestions. The tension evoked by the experience of ‘not knowing’ how to proceed towards the pre-set goal stimulated creativity and focus	*Each phase was followed by critical reflection on the aesthetics of the work. In each phase she was highly critical of her achievements and experienced frustration with not knowing if the end result would match her expectations. She was disturbed by several small setbacks while working with the material. For example, the paper mâché obscured the required transparency, and part of it had to be removed carefully, which was a delicate and frustrating job* *I chose to stimulate her experience of not knowing how to respond to the material and did not try to soften it by providing my insights on the art making process. I felt this experience of struggle would stimulate her to take appropriate action towards the material and at the same time did resemble her personal quest to deal with setbacks and slow progress. My role was to challenge her a bit by leaning back and let her come up with a response*
*Characteristic 3: Moments of uncontrollability* Uncontrollability was evident in the patient’s struggle to adhere to her own high standards regarding the art work and not knowing whether the end result would match her expectations. Uncontrollability was also induced by the way the artist let the patient create different artistic expressions over time without intervening (*Looking for the field of constant tension*). The patient’s rock changed each time until the next phase was initiated (*Expressions in a sequence*). Also, the artist supported the patient to realise that the meaning the patient attached to the work was not fixed but could change over time (*Making space to be surprised*). In combination with the high standards of the patient, the artist chose to use the art work as an object of focus in the process of supporting the patient to experience uncontrollability (*Exposing the sting of the experience of uncontrollability*), and covertly created a tension within the work relationship by not acting as a guide or leader. The artist could do this because she already had established trust and connection (*Attunement*). The artist had carefully listened to the patient’s life story (*Attunement*) and her struggles with her changing identity as she was facing death (*Exposing the sting of the experience of uncontrollability*), and allowed herself to be affected by the patient. She befriended the patient by taking a subservient role towards the patient and thus attuned to the patient’s needs (*Attunement*)	*As an artist I have tried to stay in a subservient role towards her. I went along with her struggles arising from her high standards and agreed with her decisions. Each time it felt as if she could create more space to receive the world beyond her own life struggles. I felt that investing in our relationship was the most important way to help her to deal with the many ups and downs of her current life situation (here the artist focussed on the resonance relationship between patient and artist). I was constantly checking in with her needs and longings as the work of art was changing, and I chose not to focus too much on the aesthetics of the end product. The sense of constant changeability did not leave her but we were able to handle it together*
*Characteristic 4: Adaptive transformation* At the end of the co-creation process, an adaptive transformation took place. The patient told the artist that with the creation of the work of art, she aimed to leave something of herself behind in this world, so her identity could survive beyond her physical death. Gradually her motivation extended beyond herself: to leave something behind that could be of support for her loved ones	*When we placed the light into the rock, her son was present. She had much worries about his future where she could not support him. He commented that now the rock looked like a crystal containing her energy. She was deeply moved by his comment and realized that she was not making this work of art from a place of defining her previous position in life, but that it resembled her letting go of life and being able to leave something behind to support her family*
Case two. Characteristics of resonance and emphasis on the resonance relationship between patient and material during moments of uncontrollability This 56-year-old female patient was not undergoing treatment for bile duct cancer at the time of co-creation. As part of the ISOS intervention, she had chosen to read a story called ‘Yunus’ [23], derived from the Koran, in which she identified with the protagonist Yunus who is forced to jump out of a ship and got trapped in the stomach of a whale. She felt that her illness and the accompanying struggle with her identity had befallen her and that her fate was just as undecided as it was for the protagonist of this story. Also, she was struggling with the question how she could define herself, now that she lived with cancer. This question was related to her changing body and the reaction of her environment towards her. People told her that she still looked great even at the time she was undergoing treatment. She used to feel sexy but now she felt more like a labourer and found it difficult to show others that she was not just a person who looked good, but that all elements of her being, including her illness, were allowed to be seen by herself and others and that they were of equal value	
*Characteristic 1: Being affected, touched and moved* The artist invited the patient to explore several materials and gave her books and objects to take home to see if she could connect with this input. She asked the patient to tell in detail what she felt and tried to resemble these feelings by matching material, colour, drawings and configurations	*I guess because I reached out to her as a fellow human being, and shared information about myself from the start, an even relationship was created between us. I felt that everything she would bring was important, because we could use it to make something beautiful. I gave her books and a small sculpture to take home to see whether she felt affected by them or could relate to any of them. Starting the construction of a work of art, I first had her make a drawing of her body to deepen the focus towards her relationship with her changing body. She experienced a scream in her throat that was related to her children. She felt an unbearable pain of having to die and leave them behind. She said her children are not aware of her body deteriorating and breaking down because it is not visible on the outside. That pain was visualized as either black or white smoke. I invited her to slow down every drawing movement to experience just how it affected her*
*Characteristic 2: Self-efficacy, responding* The artist tried to evoke a sense of recognition in the patient, which would lead to the patient being able to shape her inner feelings through the use of the material. To support this process, the artist and the patient looked for plant leaves to make different configurations. The aim was to see whether the patient could find the right configuration, based on an inner sense of ‘fitting’ her feelings in the configuration. In terms of choosing a colour, the same process of finding ‘the right colour’ took place	*I asked her to try to voice her pain through shaping clay and she made a shape that to her looked like a cobra, a moving shape that was feminine and protective. Then we found the shape of a certain plant that resembled the flowing movement of her inner experience. Within each phase I was mostly interested in defining the colour, form, weight and direction of movement of her inner experience. We made configurations of the plant leaves until she felt it fitted her experience. In the end we made a construction of several leaves out of steel. A pin had to be pierced right through the leaves to resemble the inner pain she was experiencing. Then we used hydrochloric acid to create stains of corrosion. We both were equally active in the creative process. She responded to the material and was able to work effectively with it, only at the end I sawed the steel myself and worked with the hydrochloric acid as this was too technical and also a bit dangerous to involve her*
*Characteristic 3: Moments of uncontrollability* The patient was in a process of redefining who she was, she did not know how to integrate all aspects of her current life into her identity and perceived to have no control over this. The artist made sure there was a high level of attunement (*Attunement*). The artist kept asking the patient ‘could all of this be you?’ and tried to make space to be open (*Making space to be surprised*) to the uncontrollability of the experience of a shifting identity (*Exposing the sting of the experience of uncontrollability*). The artist made the inner experience of the patient visible and each time a next step in the shaping of the work of art was made (*Expressions in a sequence*), the patient recognised an aspect of her experience and confirmed this by exclaiming ‘this is it!’ For the artist this confirmation was an invitation to ask for more details (*Looking for the field of constant tension*), and thus invited the next movement into the inner experience of the patient	*She would cry out “This is it, this is me!” To me this was not a moment to be seen as fixed and to dwell in it for too long, but to see it as an entry point, initiating a new movement of experimentation. To me it seemed that she was trying really hard to identify with the created shapes as if to feel safe; these shapes created shelter places, safe havens from where she was able to control her inner experience. I kept asking: “Is this you, or are you carrying this aspect, this part too?”. I asked her if she could be all these different aspects, including all differentiations occurring due to the illness, including embracing the pain, sadness and anger. I kept initiating new elements and compositions of the material to enable her to let go of the perspective she would fixate on (here the artist focussed on the resonance relationship between patient and material). Sometimes a new movement was embraced immediately, and sometimes it took longer*
*Characteristic 4: Adaptive transformation* The patient started the co-creation in a mode of trying to control her identity in relation to the threat of her changing body. However, the resulting artwork reflected her experience of uncontrollability of dealing with her changing body, which resulted in the patient embracing her illness as a given that changed her continuously and affected her identity. Adaptive transformation was also visible in the relation between the patient and her family, who at first knew very little about what the patient was going through, and in the end had learned about her vulnerability, which she was finally able to share	*I sensed that throughout the course of our meetings, she talked about her illness in a different way. At first, she would declare that she was not a palliative patient for certain, later she became somewhat yielding and talked about letting go of fighting against change, and accepting herself including the more difficult aspects of herself. Then she decided to take the work of art to her home, and shared the meaning with her family, who did not really know these things about her inner experience*

## Discussion

To the best of our knowledge, this is the first study describing that within co-creation, from the perspective of the artist, uncontrollability has a central role and showed to be multi-layered and included several distinct dynamics and mechanisms. Also, artists both actively use attunement and keep the patient interested and connected to the co-creation process during moments of uncontrollability by focussing on either or both resonance relationships. Furthermore, resonance relationships, as defined by their four characteristics, are shown to be present in co-creation.

One of the main characteristics of resonance theory, uncontrollability, plays a central role in co-creation as it affects the patient and invites responses from within the patient which can be expressed in subsequent stages of the work of art. Also, the encounters with uncontrollability can influence the perspective of the patient which can change over time. Uncontrollability also shows similarities to the element of uncontrollability in life events. These experiences of uncontrollability are sometimes referred to as experiences of contingency, which are described as having a nature to befall us unexpectedly [30]. Contingency refers to the idea that everything—including one’s own life—could have been different, one’s plans and expectations could have developed otherwise [15, 31, 32]. An experience of contingency is understood as the experience of a life event that introduces an undesired future, which threatens one’s existence, life goals and sense of meaning [1, 30, 33]. Living with cancer could be an experience of contingency which could include a high level of uncontrollability [34]. According to Zirfas [35], art is a solidification of uncontrollability, and the effect art has on the observer is uncontrollable and can differ at each subsequent encounter with the work of art. Possibly, for a patient, practising with the uncontrollability of creating art within resonance relationships in a co-creation setting could have a positive effect on the patient’s ability to successfully deal with the uncontrollability of the experience of contingency that is focussed on.

Attunement is the word artists use to describe the level of resonance within resonance relationships. Artists consciously and actively attune to the patient to establish a high intensity of resonance. Attunement illustrates that resonance not only automatically emerges but that artists consciously support the establishment of a high intensity of resonance. In addition to this, artists also actively modulate the level of attunement and subsequently the intensity of resonance in the resonance relationship between patient and both material and artist. In this way, both the intensity of the patient’s experience of working with the material and the patient’s own emotions can be influenced. In attunement, there is conscious and active participation from the part of the artist in reaching out to the patient and adapting or adjusting to the patient. Our findings on attunement connect to a larger field of research where attunement is described as a factor influencing content and outcomes of various processes, as for instance in looking at art [36], art therapy [12], counselling [37] and psychology [38]. Previously, in resonance theory, a more unconscious process of an unfolding relationship between patient and both artist and material was supposed. Our finding is in line with more recent work of Rosa in which he describes that in a classroom situation the teacher also has to actively connect with the pupils to make them interested and become affected by the world of the subject that is taught by the teacher [39]. Based on our case descriptions, it could be hypothesised that in resonance relationships between artists and patients both the level of attunement and fluctuations in these levels influence the way the patients resonate with the work of art, or can identify with the work of art. A patient’s perspective on attunement could deepen our understanding of how the resonance relationships between patient and artist, and patient and material, affect reworking experiences of uncontrollability within one’s life narrative.

Also, we found that during the co-creation process resonance relationships can provide a point of focus to alternate with moments of uncontrollability. Artists keep the patient interested and connected to the co-creation process during moments of uncontrollability by focussing on either or both resonance relationships, i.e. between patient and material, between patient and artist, or both.

Finally, within co-creation, resonance relationships between patient and both artist and material occur. These resonance relationships are defined by the four main characteristics of resonance theory: being affected, self-efficacy, uncontrollability and adaptive transformation. In our research, we found uncontrollability to be multi-layered encompassing five distinct modes of operation within co-creation. Also, artists consciously stimulate the emergence of resonance relationships. If these modes of operation and conscious creation of resonance relationships are also present in resonance relationships in other settings, this would be a further elaboration of resonance theory. As artists and their supervisors were not trained in, or familiar with resonance theory, the finding of the four main characteristics of resonance within co-creation could be seen as some form of confirmation of resonance theory as described by Rosa [21].

The total of the above-described findings could be used as a template to further unravel and understand the intricate process of co-creation.

Strengths of the study include that the artists were very experienced in their field of expertise, had previous experience in working with co-creation and had a high level of reflection on processes within themselves and in relation to the patient. Also, the involvement of artists with different fields of expertise and their ability to work with hybrid art forms contributed to creativity in the art making processes and as such positively affected artist-patient dynamics. A limitation of the study is that only the perspective of the artists is used while the perspective of the patients might be different. Thus, findings of the current study need to be confirmed by studies on the patients’ perspective.

## Conclusion

This study showed uncontrollability and attunement to be important factors in co-creation. The study confirmed the presence of resonance relationships within co-creation and showed how these resonance relationships can be used within co-creation. Focus on elements of resonance relationships within co-creation, specifically attunement and practising with uncontrollability while working with art, could strengthen interventions targeting integration of life events, i.e. experiences of contingency. Interventions using art, specifically the joint creation of a work of art, may contribute to supportive care for survivors with advanced cancer. Yet, further research to confirm this in patients and to evaluate effects on quality of life is needed.

## Data Availability

Not applicable.
